# Light quality modulates metabolic synchronization over the diel phases of crassulacean acid metabolism

**DOI:** 10.1093/jxb/eru185

**Published:** 2014-05-06

**Authors:** Johan Ceusters, Anne M. Borland, Tahar Taybi, Mario Frans, Christof Godts, Maurice P. De Proft

**Affiliations:** ^1^Faculty of Engineering Technology, Department of Microbial and Molecular systems, Bioengineering Technology TC, KU Leuven Campus Geel, Kleinhoefstraat 4, B-2440 Geel, Belgium; ^2^School of Biology, Newcastle Institute for Research on Sustainability, Devonshire Building, Newcastle University, Newcastle upon Tyne, NE1 7RU, UK; ^3^Biosciences Division, Oak Ridge National Laboratory, Oak Ridge, TN 37831-6407, USA; ^4^Faculty of Bioscience Engineering, Department of Biosystems, Division of Crop Biotechnics, KU Leuven, Willem De Croylaan 42, B-3001 Heverlee, Belgium

**Keywords:** CAM, carbohydrate, gas exchange, light quality, PEPC, PEPCK, titratable acidity.

## Abstract

Temporal compartmentation of carboxylation processes is a defining feature of crassulacean acid metabolism and involves circadian control of key metabolic and transport steps that regulate the supply and demand for carbon over a 24h cycle. Recent insights on the molecular workings of the circadian clock and its connection with environmental inputs raise new questions on the importance of light quality and, by analogy, certain photoreceptors for synchronizing the metabolic components of CAM. The present work tested the hypothesis that optimal coupling of stomatal conductance, net CO_2_ uptake, and the reciprocal turnover of carbohydrates and organic acids over the diel CAM cycle requires both blue and red light input signals. Contrasting monochromatic wavelengths of blue, green, and red light (i.e. 475, 530, 630nm) with low fluence rates (10 μmol m^–2^ s^–1^) were administered for 16 hours each diel cycle for a total treatment time of 48 hours to the obligate CAM bromeliad, *Aechmea* ‘Maya’. Of the light treatments imposed, low-fluence blue light was a key determinant in regulating stomatal responses, organic acid mobilization from the vacuole, and daytime decarboxylation. However, the reciprocal relationship between starch and organic acid turnover that is typical for CAM was uncoupled under low-fluence blue light. Under low-fluence red or green light, the diel turnover of storage carbohydrates was orchestrated in line with the requirements of CAM, but a consistent delay in acid consumption at dawn compared with plants under white or low-fluence blue light was noted. Consistent with the acknowledged influences of both red and blue light as input signals for the circadian clock, the data stress the importance of both red and blue-light signalling pathways for synchronizing the metabolic and physiological components of CAM over the day/night cycle.

## Summary

Besides the acknowledged roles of red light, blue light is a key determinant for synchronizing the metabolic and physiological components of CAM over the day/night cycle.

## Introduction

Crassulacean acid metabolism (CAM) is a photosynthetic specialization, which permits the net uptake of CO_2_ at night and thereby improves the water-use efficiency (WUE) of carbon assimilation in up to 7% of terrestrial higher plants (Crayn *et al.*, 2004). CAM plants show a remarkable metabolic plasticity for modulating nocturnal and diurnal CO_2_ uptake and have been identified as competitive biomass accumulators in comparison with many C_3_ and C_4_ crops (Borland *et al*., 2009, 2011). The diel cycle of CAM photosynthesis is commonly divided into four phases that integrate patterns of net CO_2_ uptake and changes in the abundance of key metabolites that define carbon supply and demand over the 24h cycle (Osmond, 1978). The enzyme phosphoenolpyruvate carboxylase (PEPC) is activated at night (Phase I) with the resultant uptake of CO_2_ leading to nocturnal accumulation of malic acid and a concomitant depletion in storage carbohydrates (i.e. soluble sugars or starch), which deliver the phosphoenolpyruvate (PEP) building blocks to sustain malate formation. During the day, PEPC is deactivated and malate decarboxylation mediated by phosphoenolpyruvate carboxykinase (PEPCK) or (NAD)P malic enzymes, depending on the species, releases CO_2_ within the leaf chlorenchyma cells. Consequently ribulose-1,5-bisphosphate carboxylase/oxygenase (Rubisco) fixes the CO_2_ released from decarboxylation (Phase III) behind closed stomata. These major CAM phases are punctuated by Phase II at the start of the day and Phase IV at the end of the day (Borland and Taybi, 2004). All four phases of the CAM cycle show plasticity in terms of magnitude and duration, which seems to be critical for optimizing carbon gain and water use under different environmental situations (Ceusters *et al*., 2008a, 2009a, 2009b, 2010, 2011; Dodd *et al.*, 2003).

Light intensity (photosynthetic photon flux density, PPFD) is a critical factor for determining the magnitude and duration of each of the four phases of CAM, illustrating a cardinal role for the light reactions of photosynthesis in achieving metabolic synchronization. PPFD during Phase III determines the rate of organic acid mobilization from the vacuole (Barrow and Cockburn, 1982; Thomas *et al.*, 1987), which seems to be linked to the rates of electron transport and Rubisco mediated net CO_2_ uptake *in vivo* (Lüttge, 2004). Daytime integrated PPFD also influences the magnitude of Phase I dark CO_2_ uptake by determining the abundance of carbohydrate generated via the Calvin cycle and gluconeogenesis which is subsequently required for the nocturnal provision of PEP (Nobel and Hartsock, 1983). In addition, high PPFD can elicit the induction of CAM in some facultative species that include *Guzmania monostachia* and *Clusia minor* (Maxwell *et al*., 1994, 1995; Grams and Thiel 2002). The operation of CAM is also influenced by light quality, which implies cardinal roles for certain photoreceptors in synchronizing metabolism over the diel phases. Phytochrome was shown to play a central role in the persistence of the CAM-defining circadian rhythms of CO_2_ fixation under red light as first demonstrated in *Bryophyllum fedtschenkoi* by Harris and Wilkins (1976, 1978a, 1978b). In addition, phytochrome has also been implicated in the short-photoperiod induction of CAM in *Kalanchoe blossfeldiana* (Brulfert *et al.*, 1982, 1988; Taybi *et al*., 2002). The role of blue light receptors in the operation and synchronization of the metabolic components of CAM is less clear. Previously, it was suggested that a UV-A blue light receptor mediates the high PPFD induced switch from C_3_ to CAM in *Clusia minor* (Grams and Thiel, 2002). However, no detectable effect of blue light on the persistence, phase, or period of the rhythm of CO_2_ metabolism in *Bryophyllum* leaves was noted by Wilkins (1992), implying a minor role for the blue/ultra violet A (UV-A) absorbing cryptochromes in the synchronization of CAM phases. The apparent blue-light insensitivity of CAM stomata reported for *Portulacaria afra* and *Mesembryanthemum crystallinum* has been proposed as a central component for ensuring daytime closure of stomata during phase III of CAM (Lee and Assmann, 1992, Tallman *et al.* 1997). Taken together, these observations might imply a possible C_3_ to CAM divergence in light signalling pathways mediated by blue and red light photoreceptors. Recent insights into the molecular components of the CAM circadian clock, which seem to be comparable to those of the C_3_ clock of *Arabidopsis thaliana* (Boxall *et al.*, 2005), raise new questions about the influence of light quality on orchestrating the diel cycle of CAM. Both phytochromes and cryptochromes constitute the main photoreceptors that mediate light input into the circadian clock (Hotta *et al.*, 2007). With CAM relying on a strict temporal compartmentation of metabolic processes (Borland *et al.*, 1999; Hartwell, 2005), it might be hypothesized that optimal coupling of stomatal conductance, net CO_2_ uptake, and the reciprocal turnover of carbohydrates and organic acids over the diel cycle requires both blue and red light input signals.

The aim of the present work was to examine the influence of light quality on synchronizing the phases of CAM. The obligate CAM bromeliad, *Aechmea* ‘Maya’, was exposed to different monochromatic wavelengths of light for periods of 16 hours over two complete diel cycles. By providing the different monochromatic light treatments at low fluence rates of 10 μmol m^–2^ s^–1^ using LED illumination, the intention was to minimize direct involvement of photosynthetic processes in driving CAM and thereby highlight the involvement of different photoreceptors and associated signalling pathways in metabolic synchronization. As natural daylight is composed of nearly equal amounts of red and blue light, similar quantum fluence rates were applied for these wavelengths of light. Leaf gas exchange patterns, diel gene expression analyses, protein abundance and activities of key enzymes such as phosphoenolpyruvate carboxylase (PEPC) and phosphoenolpyruvate carboxykinase (PEPCK), titratable acidities, and storage carbohydrate turnover (i.e. starch and sucrose) were monitored to examine metabolic synchronization.

## Materials and methods

### Plant material and experimental sampling

*Aechmea* ‘Maya’ is a spineless cultivar resulting from a cross between *A. tessmannii* and *A. fasciata.* These *Aechmea* species are CAM bromeliads and belong to the subfamily of the Bromelioideae (Benzing, 2000; De Proft *et al.*, 2007). Previous studies have indicated that *A.* ‘Maya’ is an obligate CAM plant (Ceusters *et al*., 2008b, 2009c, 2010). Plants were cultivated in a growth room with a daytime temperature of 20±2 °C and a minimum nocturnal temperature of 18±2 °C and PPFD of 100 μmol m^–2^ s^–1^ between 06.00 and 22.00h. Watering was performed once weekly with a conventional nutrient solution of 1 mS cm^–1^ (Londers *et al.*, 2009).

To investigate the influences of light quality on the phases of CAM, different LED (Roithner LaserTechnik, Vienna, Austria) configurations were used; i.e. blue (475±25nm), green (530±25nm) and red (630±25nm) light. At the onset of the day (06.00h) separate batches of twelve months old vegetative *Aechmea* ‘Maya’ plants (*n*=5) were placed under each configuration with a low fluence rate of 10±2 μmol m^–2^ s^–1^ and a photoperiod from 06.00–22.00h for 2 complete diel cycles. In addition, a control batch of plants was exposed to continuous darkness for 48h and a further batch of plants was exposed to white light (100 μmol m^–2^ s^–1^, photoperiod from 06.00–22.00h). Previous experiments indicated that white light of 15 μmol m^–2^ s^–1^ was insufficient to sustain metabolic activity on the short term (Ceusters *et al.*, 2011) and therefore 100 μmol m^–2^ s^–1^ was used instead of 10 μmol m^–2^ s^–1^. Leaf samples (*n*=5 plants) were taken from the upper one-third of young fully expanded leaves during the second light treatment cycle of 24h starting from 06.00h every 4h. Samples were immediately frozen in liquid nitrogen and stored at –80 °C except for the determination of enzyme activities, which were performed on fresh leaf samples. Additionally, net leaf gas exchange was also monitored for three replicate plants of each treatment.

### Gas exchange measurements

Net CO_2_ exchange was measured on the youngest fully expanded leaves, using a LCi Portable Photosynthesis System (ADC BioScientific Ltd., UK). The top part of the leaf was enclosed in a broad leaf chamber (6.25cm^2^) and the incoming air was passed through a 25 l bottle to buffer short-term fluctuations in the CO_2_ concentration. Gas exchange data were collected over a 24h period with measurements obtained at 15min intervals (*n*=3 plants). Integrated net CO_2_ uptake was determined for specific periods during the 24h time course by calculating specified areas under the CO_2_ exchange curves (Griffiths *et al.*, 1986).

### Chemical analyses of metabolites

Titratable acidity was determined by titration of methanol extracts against 1 N NaOH to a neutral endpoint, as indicated by phenolphthalein.

Soluble sugars (glucose, fructose, and sucrose) were extracted, subjected to enzymatic treatment (Enzytec, Scil Diagnostics GmbH, Germany) and analysed at 340nm using a spectrophotometer (DU-65, Beckman, Fullerton, USA). Starch content was determined as glucose equivalents following digestion with amyloglucosidase (Enzytec, Scil Diagnostics GmbH, Germany). The analyses were conducted as earlier described by Ceusters *et al.* (2008a).

### Western blotting

Approximately 250mg of powdered tissue was mixed with 250 μl of a buffer containing 100mol m^–3^ Tris, pH 8.3 at 4 °C, 10mol m^–3^ NaCl, 5mol m^–3^ ethylenediamine tetraacetic acid (EDTA), 10mol m^–3^ dithiotreitol (DTT) plus 4 μg leupeptin, 4 μg E-64, 2mol m^–3^ phenylmethanesulfonyl fluoride (PMSF) and 20 μl of a plant protease inhibitor cocktail (all protease inhibitors from Sigma, UK). The extract was centrifuged at 4 °C for 10min at 12,000 *g* and the supernatant was mixed thoroughly with glycerol (10% final volume). Protein contents were determined as described by Bradford (1976). Exactly 15 μg protein was resolved on 12% polyacrylamide gels and blotted onto polyvinyldene fluoride membranes (Sigma, UK). PEPC abundance was determined using antibodies raised against the purified enzyme from *Panicum miliaceum* (gift from Prof H. Bohnert) and PEPCK with antibodies against the purified enzyme from *Panicum maximum* (gift from RP Walker). Anti-IgG alkaline phosphatase conjugate was used as the secondary antibody. Detection was achieved using the enhanced chemi-luminescence (ECL) system (Amersham, Buckinghamshire, UK) following manufacturer’s instructions. For all blots, duplicate gels were stained with Coomassie Blue to confirm equal loading of the samples and protein integrity.

### Enzyme activities

The extraction and assay of PEPC was based on the method described by Borland and Griffiths (1997). Exactly 0.6g leaf material was homogenized in 3ml extraction buffer at 4 °C containing 200mM Tris-HCl (pH 8.0), 2mM EDTA, 1mM DTT, 2% (w/v) PEG 20 000, 1mM benzamidine, and 10mM malate with 60mg sodium bicarbonate. The homogenate was filtered through three layers of muslin and centrifuged for 2min at 13000 *g*. The extract was then desalted in a column of Sephadex G-25, equilibrated with 100mM Tris-HCl (pH 7.5 at 4 °C), 1mM DDT, 1mM benzamidine, and 5 % (w/v) glycerol. The maximal activity of PEPC was assayed in a reaction mix containing, in 1 ml: 50mM Tris-HCl (pH 8.0), 5mM MgCl_2_, 0.2mM NADH, 10mM NaHCO_3_, and 2.5mM PEP. The reaction was initiated by the addition of 50 μl of extract and change in absorbance at 340nm was measured for 4min at 25 °C.

The extraction and assay of PEPCK was adapted from the methods described by Cooper *et al.* (1968) and Walker and Leegood (1995). Exactly 0.3g leaf tissue was homogenised at 4 °C with 100mg PVPP in 3ml extraction buffer containing 50mM Hepes-NaOH (pH 7.7), 1mM EDTA, 5mM Mg-acetate, 1% (w/v) bovine serum albumin, 5mM DTT, and 1mM PMSF. The homogenate was centrifuged for 1min at 13 000rpm at 4 °C and 75 μl of the supernatant was used immediately in a reaction mix containing, in 1 ml: 40mM Mes-KOH (pH 6.7), 4.2mM glutathione reduced, 0.35mM NADH, 1mM MnCl_2_, 50mM KHCO_3_, 2mM ADP, 2mM PEP, and 6 units malate dehydrogenase (Sigma Aldrich). To determine the maximal activity of PEPCK the change in absorbance at 340nm was measured for 4min at 25 °C. Similar reactions without ADP or PEP were carried out to eliminate potential background reactions catalysed by PEPC or pyruvate kinase plus lactate dehydrogenase (Hatch, 1973).

### RNA extraction and semi-quantitative RT-PCR

Total RNA was purified from 250mg of powdered leaf tissue using Tri-Reagent (Helena Biosciences, UK) as described by Taybi *et al.*, (2000). Exactly 5 μl of RNA extract was treated with DNase I (Life Technologies, Paisley UK), to prevent amplification of genomic DNA. RT-PCR was conducted as a single-tube reaction and cDNA synthesis was promoted using the reverse primer. Each 25 μl reaction contained 10x PCR reaction buffer, 10mol m^–3^ DTT, 2.5mol m^–3^ MgCl_2_, 0.25mol m^–3^ dNTPs (Bioline Scientific, London, UK), 400nM each of forward and reverse primers, 12U RNase Out (Life Technologies, UK), 0.5U Taq DNA polymerase (Bioline Scientific), 30U Superscript II reverse transcriptase (Life Technologies) and 100ng total RNA. Annealing temperature was 50 °C and 25 and 30 cycles were used for amplification of *ppc* and *pepck* respectively. The following primers were used: PPC F (5’ GTG GGA CTG TGG GGA GA 3’), PPC R (5’ CTT GTC TGT GTC CAC GCA 3’), PEPCK F (5’ CAC GCC TGA AGA GCT AG 3’), PEPCK R (5’ CAG GGC ATC TTT GCG TTA 3’). Ubiquitin served as internal control as well as control for equal RNA inputs and RT-PCR conditions. To confirm specificity of RT-PCR, the PCR products obtained from using the *ppc* and *pepck* primers were sequenced and confirmed against publicly available sequences for the two genes using blast. The partial sequences obtained for *ppc* and *pepck* from *A. ‘maya*’ were deposited on the National Center for Biotechnology Information (NCBI; KC007431 for *ppc* and KC007432 for *pepck*).

### Data analysis

Where appropriate, data were analysed using the statistical software package SAS Enterprise Guide 4.0. Before carrying out statistical tests, normality of the data was checked by means of the Kolmogorov-Smirnoff statistic (p>0.05). Means are compared either by two sample t-test or Tukey’s studentized range test (α=0.05).

## Results

### Leaf gas exchange

**Fig. 1.**
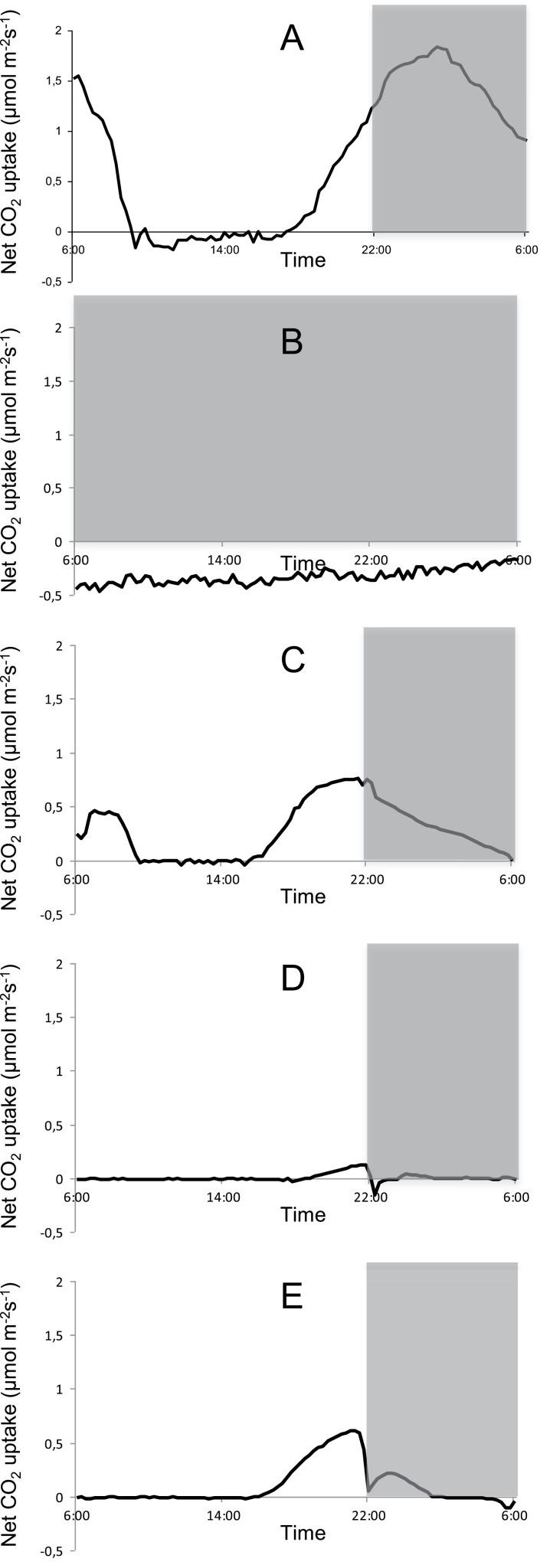
Net 24h CO_2_ uptake (μmol m^–2^ s^–1^) for young fully developed leaves of *Aechmea* ‘Maya’ under control light-dark cycle (A, white light, 100 μmol m^–2^ s^–1^), continuous dark (B), and different monochromatic light-dark cycles (10 µmol m^–2^s^–1^; C=blue 475nm; D=green 530nm; E=red 630nm). The dark period is indicated in grey. Gas exchange curves are representative of three replicate runs with SE<15%.

**Fig. 2.**
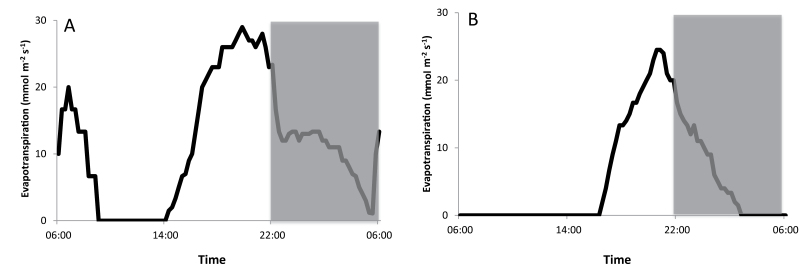
Net 24h evapotranspiration (mmol m^–2^s^–1^) for young fully developed leaves of *Aechmea* ‘Maya’ under blue (A) and red (B) illumination of 10 μmol m^–2^ s^–1^. The dark period is indicated in grey. Curves are representative of three replicate runs with SE<15 %.

For each of the investigated wavelengths together with continuous dark and white light/dark conditions, diel gas exchange patterns were monitored and integrated net CO_2_ uptake was calculated for each of the different CAM phases (Fig. 1, Table 1). In contrast to the white light/dark control where a complete 24h CAM cycle occurred with a total diel uptake of 61.1±9.4 mmol CO_2_ m^–2^, under continuous darkness sustained respiration of CO_2_ resulted in a net loss of 28.4±4.9 mmol CO_2_ m^–2^ over 24h. Under low fluence rates of blue light (10 μmol m^–2^ s^–1^) all four phases of CAM were expressed and approximately 33% of CO_2_ was captured over 24h in comparison with the white light/dark control treatment. Afternoon net CO_2_ uptake (Phase IV) was unaffected (relative to that under white light; *P*>0.05) but night-time net CO_2_ uptake was significantly curtailed compared with that noted under the usual day/night conditions (*P*<0.05). Under low-fluence red light, 24h net CO_2_ uptake represented ~16.5% of that measured in the white light control. About 90% of total net CO_2_ uptake under red light occurred during the afternoon (Phase IV) with no Phase II and a restricted Phase I. The abolishment of an early day stomatal opening for red illuminated plants, although clearly present for blue illuminated ones, was further confirmed by leaf evapotranspiration data for blue and red illumination (Fig. 2). Under low-fluence green light a small but significant (*P*<0.05) amount of net CO_2_ uptake (1.1±0.5 mmol CO_2_ m^–2^) during a shortened Phase IV was noted but no net respiration was recorded over the diel cycle in contrast to that observed under continuous darkness (Fig. 1, Table 1).

**Table 1.** Integrated net CO_2_ uptake for each of the four CAM Phases (mmol CO_2_ m^–2^ Phase^–1^) by young fully developed leaves of *Aechmea* ‘Maya’ under control light-dark cycle (LD, white light, 100 μmol m^–2^ s^–1^), continuous dark (DD) and different monochromatic light-dark cycles (10 μmol m^–2^s^–1^) Data are means±SE (*n*=3) and significant net CO_2_ uptake/loss (*P*<0.05) is indicated by an asterisk

	Phase II	Phase III	Phase IV	Phase I	Total 24 h
LD	11.1±2.5*	–2.0±1.8	9.9±3.2*	42.8±5.4*	61.1±9.4*
DD	N/A	N/A	N/A	N/A	–28.4±4.9*
Blue	3.9±0.5*	0.1±0.8	9.2±1.7*	9.9±1.9*	23.1±3.4*
Green	–0.1±0.2	–0.2±0.2	1.1±0.5*	0.1±0.2	0.8±0.5*
Red	–0.1±0.1	–0.2±0.3	7.5±1.3*	1.3±0.4*	8.5±1.7*

### Metabolite dynamics

**Fig. 3.**
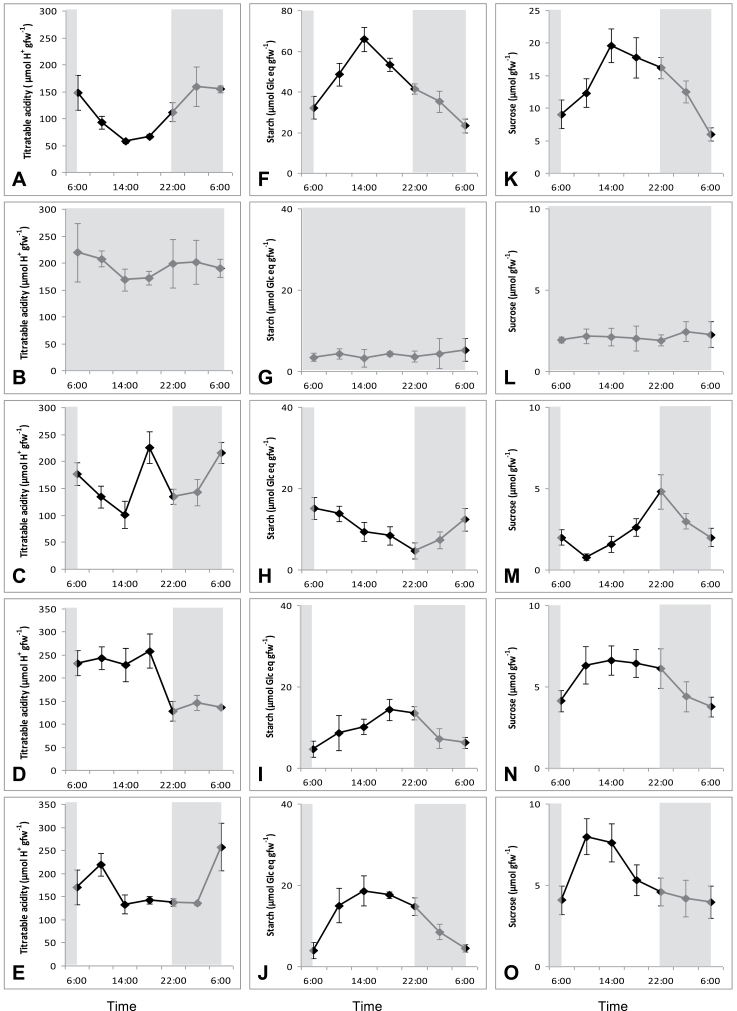
Diel patterns of titratable acidity (μmol H^+^ g^–1^fw; left panel), starch (μmol Glc eq g^–1^fw; middle panel) and sucrose (μmol g^–1^fw; right panel) for young fully developed leaves of *Aechmea* ‘Maya’ under control light-dark cycle (A, F, K; white light, 100 μmol m^–2^ s^–1^), continuous dark (B, G, L), and different monochromatic light-dark cycles (10 μmol m^–2^ s^–1^), i.e. blue (C, H, M), green (D, I, N), and red (E, J, O). The dark period is indicated in grey. Data are means±SE (*n*=5 plants).

The typical CAM pattern of nocturnal accumulation and daytime degradation of organic acids was clearly present under white light/dark conditions (Fig. 3A). This diel cycle of organic acids was lost in continuous darkness where titratable acidities remained at maximal levels during the diel cycle (Fig. 3B). The monochromatic low-light treatments each provoked specific responses in the diel acidity cycle. Blue light induced degradation of organic acids (176±21 μmol H^+^ g^–1^ fw to 101±20 μmol H^+^ g^–1^ fw) at the start of the photoperiod in a comparable way to the plants under white light (Fig. 3C). However in the middle of the day (14.00h) titratable acidity suddenly increased to a maximal level of 223±32 μmol H^+^ g^–1^ fw followed by an immediate decline at 18.00h. During the night following blue light treatment, the organic acid content increased to levels comparable to that in the control light/dark treatment. However, although most acids accumulated over the first half of the dark period in the control white light plants, under blue light, most acid accumulation occurred in the latter half of the night. Plants subjected to red and green wavelengths showed a delay in acid breakdown at the start of the day, i.e. 4h for red and 12h for green after dawn (Fig. 3D, E). Maximal acidities in the white light control treatment noted at 02.00h corresponded with minimal values of titratable acidity at this time under green and red light Whilst there was no significant nocturnal accumulation of acids under green light (*P*>0.05), some 110±32 μmol H^+^ g^–1^ fw organic acids were accumulated overnight following red light illumination. As with the blue light treatment, nocturnal acid accumulation occurred over the latter half of the night after the red light treatment.

Under white light/dark conditions, both starch and sucrose showed an inverse diel rhythm compared with titratable acidity with starch being the main carbohydrate degraded at night to sustain nocturnal CO_2_ uptake (diel turnover of 40±6 μmol Glc eq g^–1^ fw from starch versus 20±5 μmol Glc eq g^–1^ fw for sucrose) (Fig 3, middle and right panels). In line with the titratable acidity measurements, continuous darkness abolished any diel fluctuations in storage carbohydrates and minimal values of 4±1 μmol Glc eq g^–1^ fw and 3±1 μmol g^–1^ fw persisted in the leaves for starch and sucrose, respectively. Green and red light treatments resulted in nocturnal starch degradation of 12±3 μmol Glc eq g^–1^ fw, representing 33% of starch degradation in control plants. In contrast, exposure to blue light resulted in an inverse rhythm of starch turnover with starch degradation occurring during daytime followed by nocturnal accumulation. The diel turnover of sucrose under blue, green, or red light treatments was about 33% in comparison with control plants (8±2 μmol Glc eq g^–1^ fw). The diel pattern of sucrose turnover was generally less affected by light quality as compared with that of starch. The dissacharide accumulated during the day under all light treatments but a consistent delay in sucrose accumulation occurred under the blue light treatment.

### Transcript abundance, protein content, and activities of PEPC and PEPCK

**Fig. 4.**
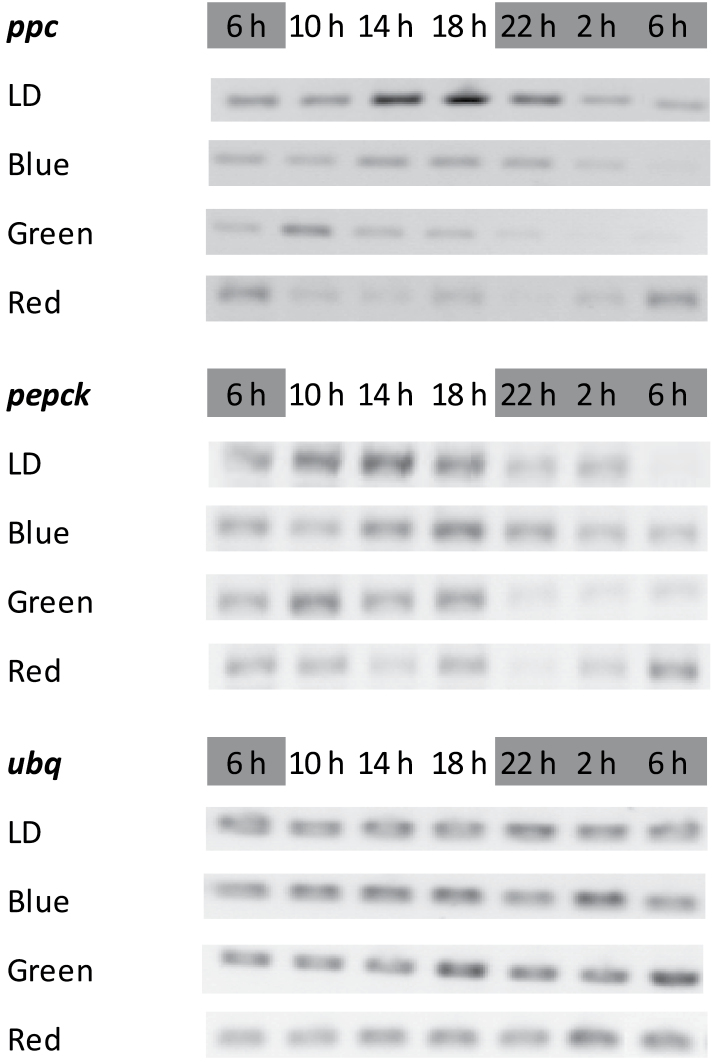
Day-night changes in levels of transcripts for PEPC (*ppc*) and PEPCK (*pepck*) in young fully developed leaves of *Aechmea* ‘Maya’ under control light-dark cycle (LD, white light, 100 μmol m^–2^ s^–1^) and different monochromatic light-dark cycles (10 μmol m^–2^ s^–1^), i.e. blue, green, and red. The dark period was from 22.00–06.00h. Ubiquitin (*ubq*) served as control.

Under white light/dark conditions a diel rhythm in transcript abundance of *ppc* was observed with maximum transcript abundance noted over the latter part of the photoperiod (Fig. 4). This rhythm in *ppc* abundance was damped under the different light treatments and except for blue illumination, the timing of maximum *ppc* transcript abundance was also shifted relative to that noted under white light. Under green light, timing of max *ppc* abundance occurred at least 6h earlier than that under white light and under red light the diel rhythm of *ppc* transcript abundance with peaks in the early morning, was almost opposite to the one under white light. The transcript abundance of *pepck* also showed a diel rhythm with highest abundance during the day under all light treatments. The green light treatment resulted in a strong decline in *pepck* transcipts at night, compared with the other treatments.

**Fig. 5.**
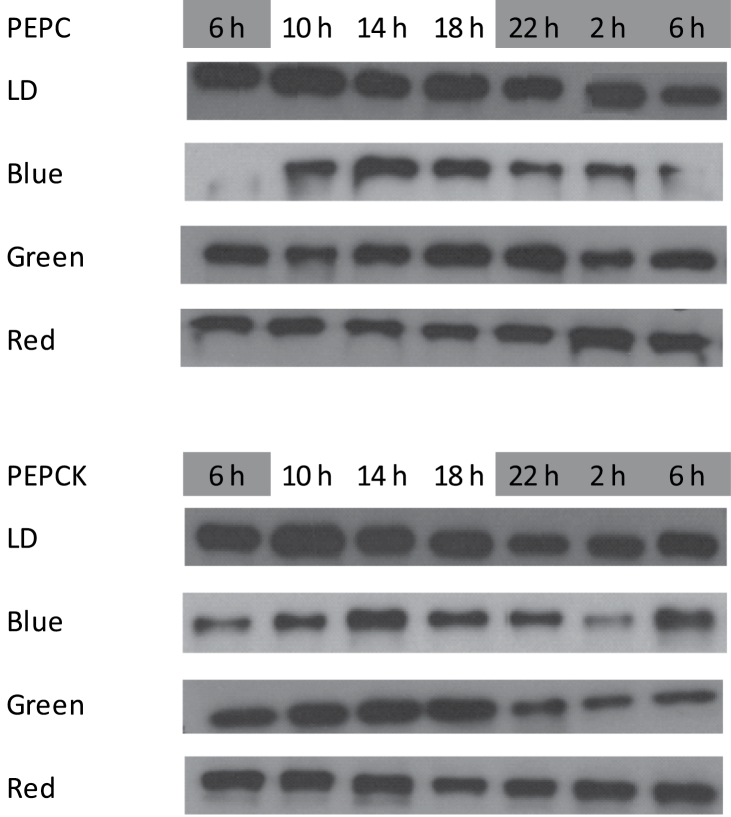
Western blots showing the abundance of PEPC (top) and PEPCK (bottom) protein in young fully developed leaves of *Aechmea* ‘Maya’ under control light-dark cycle (LD, white light, 100 μmol m^–2^ s^–1^) and different monochromatic light-dark cycles (10 μmol m^–2^ s^–1^), i.e. blue, green, and red. The dark period was from 22.00–06.00h. Similar amounts of soluble proteins were loaded for each treatment.

In terms of protein abundance, there was no obvious day/night change in PEPC protein under light/dark conditions (Fig. 5). However, under blue light, the amount of PEPC protein was reduced compared with that under white light and a diel pattern of abundance was apparent, with a decline in PEPC abundance over the night following blue light treatment. Red light also resulted in a decrease in PEPC protein. The abundance of PEPCK protein was highest under the white light treatment. Blue and red light resulted in a general decline in the amount of PEPCK protein. Under green light, the decline in PEPCK protein (compared with the white light control) was most apparent during the night period.

To quantify differences in enzyme capacities under the different light treatments the maximal activities of PEPC and PEPCK were measured with samples taken in the middle of the night (PEPC) and middle of the day (PEPCK) day (Table 2). There was no significant difference between PEPC activities in plants maintained under white (control) or blue light but red and green light resulted in a significant (*P*<0.05) decrease in PEPC activity compared with controls. The activities of PEPCK were comparable for all investigated wavelengths except for the green light treatment where PEPCK activity was twice as high (*P*<0.05) compared with the other treatments. Under continuous dark, both PEPC and PEPCK activities were significantly lower (*P*<0.05) in comparison with the light control and the monochromatic light treatments.

**Table 2.** Activities of PEPC and PEPCK (μmol CO_2_ g^–1^fw h^–1^) extracted from young fully developed leaves of *Aechmea* ‘Maya’ in the middle of the night (PEPC) and midday (PEPCK) under control light-dark cycle (LD, white light, 100 μmol m^–2^s^–1^), continuous dark (DD) and different monochromatic light-dark cycles (10 μmol m^–2^s^–1^) Data are means±SE (*n*=3) and those in each column followed by a different letter are significantly different by Tukey’s Studentized range test (*P*< 0.05).

	PEPC		PEPCK	
LD	52±14	A	33±4	B
DD	8±1	C	23±6	C
Blue	37±11	A	30±6	B
Green	23±2	B	60±4	A
Red	19±3	B	28±5	B

## Discussion

Given the nature and importance of the circadian clock for the functioning of CAM (Boxall *et al.*, 2005), the aim of the present study was to test the hypothesis that both red and blue light signalling processes are of pivotal importance for optimal synchronization of the diel phases of CAM. By building on the acknowledged influence of red light on the physiology and biochemistry of CAM plants (Wilkins, 1992), this study sought to get blue light responses ‘out of the dark’ in CAM research by comparing fundamental metabolic characteristics under diel light/dark cycles of different monochromatic wavelengths (i.e. blue, red, and green) in comparison with a control white light/dark treatment.

### Leaf gas exchange

Under a low fluence rate of 10 μmol m^–2^ s^–1^, blue light sustained a typical CAM gas exchange pattern in leaves of *A.* `Maya` consisting of all four phases of CAM (Osmond, 1978). Although total net uptake of CO_2_ was only 33% of that measured in control plants (white light, 100 μmol m^–2^ s^–1^), no major differences in the timing of the different phases of gas exchange were apparent under blue or white light. These data indicate the need for a review of the previously proposed insensitivity of CAM stomata to blue light, inferred from studies with individual leaves or isolated stomata from facultative CAM plants (Lee and Assmann, 1992; Mawson and Zaugg, 1994; Tallman *et al.*, 1997). The present study is the first in which whole CAM plants were monitored under full diel cycles. The occurrence of a complete Phase II under both white and low-fluence blue light, although absent under the other wavelengths (i.e. red and green), is consistent with the generally accepted view of blue-light induced early morning stomatal opening (Doi *et al.*, 2004; Lawson, 2009). These observations are further strengthened by early morning (i.e. Phase II) patterns of leaf evapotranspiration for blue and red illuminated plants, which clearly illustrate a qualitative light effect on stomatal control. Phase IV CO_2_ assimilation was very similar for the white light control as well as for blue and red illuminated plants. Blue light might bring about higher energy dissipation, whereas more efficient energy capture could occur under red light. Further measurements of Phase IV photosynthetic rates under different intensities of blue and red light would be informative. In summary, the CAM phases seem to show some degree of plasticity in response to light quality but the observed phenomena clearly stress the importance of blue light inputs to mediate Phase II CO_2_ uptake and sustain a significant proportion of nocturnal carboxylation (i.e. Phase I).

### Carboxylation and decarboxylation events

At the start of the photoperiod (06.00h) under low-fluence blue light, decarboxylation of malate was initiated and titratable acidities decreased to minimal values around the middle of the photoperiod (14.00h) comparable to the kinetics in control plants. In contrast, the higher wavelengths of the spectrum caused a consistent delay in breakdown of organic acids, which occurred ~4 and 12h into the photoperiod for red and green light, respectively. Comparing the maximum enzyme activity of PEPCK measured *in vitro* with measured rates of acid breakdown *in vivo* indicated that this delay in net acid breakdown was unlikely to be a consequence of insufficient intrinsic decarboxylating capacity but may have been due to postponement of malate efflux out of the vacuole. The maximum PEPCK activity measured *in vitro* was similar for all treatments except for plants exposed to green light, where a doubled activity was present that was not mirrored by changes in transcript or protein abundance. These observations suggest an influence at the post-translational level, implicating a higher degree of dephosphorylation brought about by green illumination, which could increase the maximum enzyme activity. PEPCK has been described previously to undergo reversible phosphorylation with the phosphorylated enzyme present at night when decarboxylation should be curtailed (Walker and Leegood, 1996).

Under low-fluence blue light during the latter part of the photoperiod, synchronization between carboxylation and decarboxylation events seemed to be uncoupled as premature PEPC activity was indicated by a significant net accumulation of titratable acids, followed by a 2h period of significant acid breakdown before entering the dark period. The diel oscillations of *ppc* and *pepck* transcript abundance noted in control plants were maintained under blue light but were damped in abundance. No difference was detected in maximum *in vitro* activities of PEPC and PEPCK under white or blue light. However, *in vivo* activity of PEPC is modulated by reversible phosphorylation by a dedicated kinase (PPCK), which is believed to be under circadian control at the transcript level (Hartwell *et al*., 1996, 1999; Nimmo, 2000). Moreover, it has been postulated that the same kinase could potentially also phosphorylate PEPCK *in vivo* and thereby curtail futile cycling between malate synthesis and breakdown (Dodd *et al.*, 2002). Previous studies have shown that blue light (as compared with white or red light) is particularly important for stabilizing the clock protein ZTL, thereby allowing daytime degradation of TOC1, a core clock protein that normally accumulates during the day (Fujiwara *et al.*, 2008; Harmer, 2009). Such blue light influences on core clock proteins might have profound influence on the transcript abundance of *ppck*, which is generally down regulated during the day (Taybi *et al.*, 2004). However as *ppck* has not yet been identified in a CAM bromeliad (including *Ananas comosus* (pineapple) or *A.’Maya’* (J Delahunty, J Ceusters and A Borland unpublished observations), this hypothesis remains to be tested.

### Storage carbohydrate turnover

One of the key characteristics in CAM plants is the intimate reciprocal relationship between the diel cycling of organic acids and their storage carbohydrate counterparts, which can be either soluble sugars or starch (Borland and Taybi, 2004). In line with the retention of high backgrounds of titratable acidity, the diel cycles of accumulation/degradation of starch and sucrose, which sustain PEPC carboxylation in Bromelioideae (Antony *et al.*, 2008; Ceusters *et al.*, 2008a) were abolished under continuous darkness. Similar observations were previously made for *Aechmea* ‘Maya’ under low intensities of about 15 μmol m^–2^ s^–1^ white light (PPFD 0.46mol photons m^–2^ d^–1^) (Ceusters *et al.*, 2011). However, low-fluence illumination of 10 μmol m^–2^ s^–1^ with specific wavelengths of light provoked different responses in the kinetics of storage carbohydrate turnover. Although damped in comparison with control plants, low-fluence red light assured appropriate diel cycling for both starch and sucrose, showing accumulation during the first part of the day (6–14h) followed by degradation during the second part of the day and night. As carbohydrate responses to green and red light were similar to those under white light, both red and green light might be regarded as positive regulators in inducing starch breakdown towards the end of the light period. Under blue light, sucrose accumulation was maintained until the end of the photoperiod, but a complete reversal of the starch cycle was observed with significant breakdown of starch during the day followed by nocturnal accumulation. Such a reversal of the diel starch cycle has not previously been reported in photosynthetic mesophyll cells. It remains to be established if the signals that orchestrate leaf starch turnover over the day/night cycle have diverged between the C_3_ and CAM pathways. However, blue-light-mediated degradation of starch (often accompanied by sucrose accumulation) typically resembles the response of guard cells in C_3_ plants such as *Arabidopsis* and *Vicia* when stomatal aperture increases following the onset of the light period (Tallman and Zeiger, 1988; Lasceve *et al.*, 1997). The reversal of diel starch turnover invoked by blue light in leaves of the CAM species *A. ‘maya’* is intriguing in light of previous suggestions that CAM may have evolved from C_3_ guard cell metabolism (Cockburn, 1981).

In conclusion, the results of this study indicate the importance of a combined input of different wavelengths of light to orchestrate and synchronize the diel phases of CAM. Although low-fluence blue light was a key determinant in inducing daytime decarboxylation and regulating stomatal responses, low-fluence red light was required to synchronize the diel cycles of storage carbohydrate (starch and sucrose) accumulation and degradation with acid accumulation. These novel findings open up several new avenues of research into how light quality and quantity interact to modulate the CAM phases. Further detailed experimentation is now possible and recommended, including combined inputs of both red and blue light as well as irradiance dependence curves for different light qualities to reveal more about the complex interplay of different signals that coordinate stomatal conductance with the metabolic processes that underpin CAM.
